# The characteristics and clinical outcomes of atrial fibrillation patient in middle income country of Indonesia

**DOI:** 10.3389/fcvm.2026.1824720

**Published:** 2026-07-01

**Authors:** Yoga Yuniadi, Putra S. Antara, Muzakkir Amir, Dicky A. Hanafy, Sunu B. Raharjo, Dony Y. Hermanto, M. Yamin, Hauda El-Rasyid, Benny Setiadi, Erika Maharani, Agus Harsoyo, Anggia C. Lubis, Pipin Ardhianto, Aruman Y. A. Binarso, Ardian Rizal, Rerdin Julario, Agung F. Chandranegara, Yoga Waranugraha, Chaerul Ahmad, Mohammad Iqbal, Ridwan Rasyid, Antonia A. Lukito, Haryadi Haryadi, Margono G. Suwandi, Alexander E. Tondas, Indah A. Putri, Beny Hartono

**Affiliations:** 1 Department of Cardiology and Vascular Medicine, Faculty of Medicine, Universitas Indonesia, Jakarta, Indonesia; 2 Department of Cardiology and Vascular Medicine, Faculty of Medicine, Universitas Udayana, Denpasar, Indonesia; 3 Ngoerah General Central Hospital, Denpasar, Bali, Indonesia; 4 Department of Cardiology and Vascular Medicine, Faculty of Medicine, Universitas Hasanuddin, Makassar, Indonesia; 5 Wahidin Soerodihoesodo General Central Hospital, Makassar, Indonesia; 6 National Cardiovascular Center Harapan Kita, Jakarta, Indonesia; 7 Cipto Mangunkusumo General National Hospital, Jakarta, Indonesia; 8 Department of Cardiology and Vascular Medicine, Faculty of Medicine, Universitas Andalas, Padang, Indonesia; 9 M Djamil General Central Hospital, Padang, Indonesia; 10 Department of Cardiology and Vascular Medicine, Faculty of Medicine, Universitas Sam Ratulangi, Manado, Indonesia; 11 Kandau General Central Hospital, Manado, Indonesia; 12 Department of Cardiology and Vascular Medicine, Faculty of Medicine, Universitas Gajah Mada, Yogyakarta, Indonesia; 13 Sardjito General Central Hospital, Yogyakarta, Indonesia; 14 Gatot Subroto Army General Hospital, Jakarta, Indonesia; 15 Department of Cardiology and Vascular Medicine, Faculty of Medicine, Universitas Sumatra Utara, Medan, Indonesia; 16 Adam Malik General Central Hospital, Medan, Indonesia; 17 Department of Cardiology and Vascular Medicine, Faculty of Medicine, Universitas Diponegoro, Semarang, Indonesia; 18 Kariadi General Central Hospital, Semarang, Indonesia; 19 Department of Cardiology and Vascular Medicine, Faculty of Medicine, Universitas Brawijaya, Malang, Indonesia; 20 Syaiful Anwar General Central Hospital, Malang, Indonesia; 21 Wava Husada Hospital, Malang, Indonesia; 22 Department of Cardiology and Vascular Medicine, Faculty of Medicine, Universitas Airlangga, Surabaya, Indonesia; 23 Soetomo General Central Hospital, Surabaya, Indonesia; 24 Pasar Rebo District Hospital, Jakarta, Indonesia; 25 Universitas Brawijaya Hospital, Malang, Indonesia; 26 Department of Cardiology and Vascular Medicine, Faculty of Medicine, Universitas Padjadjaran, Bandung, Indonesia; 27 Hasan Sadikin General Central Hospital, Bandung, Indonesia; 28 Hasna Medika Heart Specialty Hospital, Cirebon, Indonesia; 29 Siloam Lippo Village Hospital, Tangerang, Indonesia; 30 Eka Hospital, Pekanbaru, Indonesia; 31 Suhardi Hardjolukito Air Force Central Hospital, Yogyakarta, Indonesia; 32 Mohammad Husin General Central Hospital, Palembang, Indonesia; 33 National Brain Center, Jakarta, Indonesia; 34 Binawaluya Heart Hospital, Jakarta, Indonesia

**Keywords:** atrial fibrillation, Indonesia, middle income country, optimal INR, warfarin

## Abstract

**Background:**

Atrial fibrillation (AF) is an increasing health concern in Asia, but data from developing countries remain scarce. Although direct oral anticoagulants (DOACs) are now preferred globally, vitamin K antagonists (VKAs) remain the mainstay of stroke prevention in low- and middle-income countries, including Indonesia. This study evaluates the clinical outcomes of Indonesian AF patients with warfarin-based treatment.

**Methods:**

The OPTIMA (Optimal target of INR for Indonesian) study is a multicenter, prospective cohort study involving 1,599 AF patients across 25 centers in Indonesia. Clinical characteristics, INR levels, and time in therapeutic range (TTR) were analyzed. The primary endpoints were stroke/systemic embolism (efficacy) and major bleeding (safety). Cox proportional hazards regression and Kaplan–Meier analysis assessed outcomes.

**Results:**

The mean CHA_2_DS_2_-VASc score was 2.43. Anticoagulation quality was poor under the standard INR target of 2.0–3.0, with a mean time in therapeutic range (TTR) of only 30.1%. Patients with an average INR of 1.6–2.5 experienced lower rates of stroke and major bleeding. The incidence rates per 100 patient-years were 1.32 for stroke, 1.51 for major bleeding, and 3.15 for mortality. Stroke risk was highest in persistent/permanent AF, with younger, working-age individuals disproportionately affected.

**Conclusions:**

Warfarin management at the standard INR target of 2.0–3.0 was associated with poor anticoagulation quality. Patients with average INR values of 1.6–2.5 experienced fewer adverse events, although this finding requires confirmation in prospective trials. Socioeconomic burden is substantial, as AF-related stroke disproportionately affects younger, working-age Indonesians.

## Graphical Abstract

**Figure:**
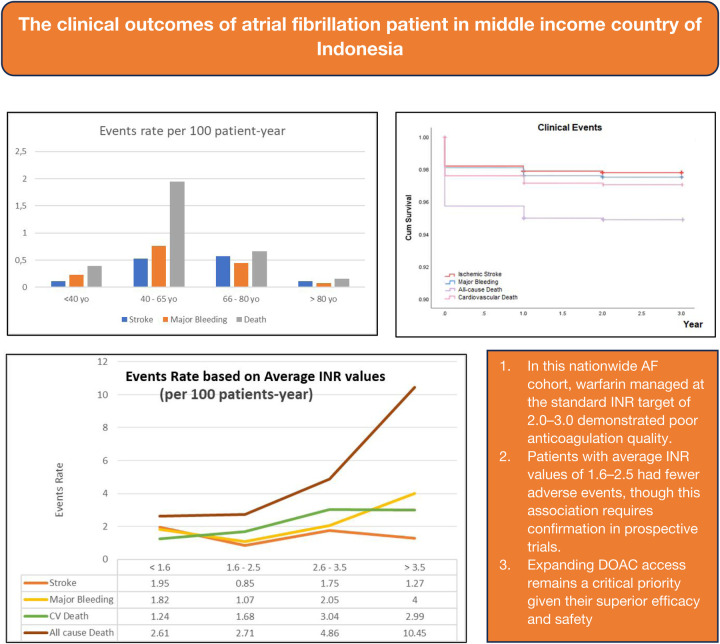

## Background

Atrial fibrillation (AF) is the most common arrhythmia encountered in clinical practice and a major risk factor for ischemic stroke and mortality (1, 2). While the prevalence of AF in Asian populations is slightly lower than in Western countries, the overall burden is significantly greater due to the large population size of Asia. By 2050, AF is projected to affect approximately 49 million men and 23 million women across Asia (3–5). The prevalence of AF has been documented in various Asian countries, including China (3, 6), Japan (7), Korea (8), Taiwan (3), Singapore (9), and Malaysia (10). However, data on AF prevalence and management in Indonesia remain limited. No large-scale prospective cohort study has been conducted on AF patients in Indonesia, and prior research has not adequately represented the country's population. A recent cross-sectional study using the AliveCor™ smartphone-based electrocardiogram system in a tertiary hospital suggested that AF prevalence in Indonesia may be higher than in other Southeast Asian nations (11).

Oral anticoagulation is the cornerstone of stroke prevention in AF management (12, 13). The direct oral anticoagulants (DOACs) are generally preferred over vitamin K antagonist (VKAs) due to their superior efficacy and safety profiles. In many developed Western and Asia-Pacific countries, the use of anticoagulants has shifted from warfarin to DOACs (14, 15). However, in several Asian countries, warfarin remains the most commonly used anticoagulant due to its affordability and availability (16).

Despite its widespread use, warfarin poses significant safety concerns, particularly for Asian populations. Studies have shown that Asians have a twofold increased risk of intracerebral hemorrhage compared with Caucasians (17). This risk rises to fourfold when warfarin is used at similar anticoagulation intensities. In Indonesia, warfarin is the only oral anticoagulant reimbursed by the National Health Insurance program, restricting access to DOACs for patients who might benefit from them.

Furthermore, genetic variations among Asian populations influence warfarin metabolism and response. Polymorphisms in the CYP2C9 gene and vitamin K epoxide reductase complex 1 (VKORC1) gene affect optimal warfarin dosing and therapeutic outcomes (18, 19). Similar gene polymorphisms have been observed in the Indonesian population, correlating with warfarin dose stability (20).

Considering these clinical factors, we conducted a prospective cohort study to elaborate clinical characteristics and evaluate the safety and efficacy of warfarin in AF patients across major cities in Indonesia. This study aims to provide real-world evidence to guide anticoagulation management strategies and optimize stroke prevention in Indonesian AF patients.

## Methods

### Patients

The study was approved by Ethics Review Board of Research and Development Bureau Ministry of Health, Republic of Indonesia. All methods were performed in accordance with the relevant guidelines and regulations. The study conducted from January 2021 up to December 2023. AF patients aged 18–80 years receiving warfarin therapy were enrolled from referral hospitals in major Indonesian cities. Patients were recruited from referral hospitals across major islands of the Indonesian archipelago to ensure broad representation. Baseline clinical characteristics, ECG and laboratory were reviewed. Informed consent was obtained from all participants. Due to remain high prevalence of Rheumatic Heart Disease (RHD), patient with AF and valvular heart disease were not excluded.

### Endpoints

The end point of efficacy (stroke and systemic thromboembolism) and safety (bleeding) were observed during 1 year follow-up. Bleeding criteria based on International Society of Thrombosis and Hemostasis (ISTH) (21) which was divided a non-surgical bleeding into major and minor bleeding. The major bleeding comprised of (1) fatal bleeding; (2) symptomatic bleeding in a critical area or organ, such as intracranial, intraspinal, intraocular, retroperitoneal, intraarticular or pericardial, or intramuscular with compartment syndrome; and (3) Bleeding causing a fall in hemoglobin level of 2 g/dL (1.24 mmol/L) or more, or leading to transfusion of two or more units of whole blood or red cells. The minor bleeding comprised of all non-major bleeds which further divided into those that are clinically relevant and those that are not.

### International normalized ratio (INR)

The International Normalized Ratio (INR) was determined according to World Health Organization (WHO) guidelines (22). Venous blood was collected in citrate, plasma was separated, and clotting was initiated with calcium and tissue factor to measure the patient's prothrombin time (PT). PT reflects the time required for blood to clot. This value was divided by the mean PT of a healthy control population to generate a ratio. Because thromboplastin reagents vary among laboratories, each reagent is assigned an International Sensitivity Index (ISI) that quantifies its responsiveness relative to a standardized reference. To correct for this variability, the ratio of patient PT to control PT is raised to the power of the ISI, yielding the INR. This standardization enables comparability of results across laboratories worldwide. PT measurement and INR calculation were performed at each site laboratory or using point-of-care (POC) devices. Each patient underwent INR testing two to three times, with measurements spaced at least one month apart.

### Time in therapeutic range (TTR)

Time in therapeutic range (TTR) is widely accepted as a measure of the quality of anticoagulation achieved with warfarin in both clinical practice and clinical trials. The Rosendaal linear interpolation method was applied to calculate TTR in this study (23). Briefly, all INR values and their corresponding dates were collected for each patient. The therapeutic range (e.g., INR 2.0–3.0) was defined. For each pair of consecutive INR values, the time interval between them was considered, and a linear change in INR was assumed. Daily INR values within each interval were estimated by interpolation. The number of days falling within the therapeutic range was counted, and this process was repeated across the entire follow-up period. The sum of days in range was divided by the total number of days observed, and the resulting fraction was multiplied by 100 to yield the percentage of time in therapeutic range.

### Statistical analysis

All statistical analyses were performed using IBM SPSS Statistics, Version 25 (IBM Corp., Armonk, NY, USA). The correlation between INR and TTR values with clinical outcomes was assessed using the Mantel–Haenszel Chi-square test. To evaluate the effect of warfarin on outcomes over a one-year follow-up, a Cox proportional hazards regression model was applied. Kaplan–Meier survival analysis was conducted to estimate event-free survival across different INR groups, with between-group comparisons performed using the log-rank test. In addition, multivariable logistic regression analysis was used to calculate odds ratios for variables that were significant in univariate analysis, including clinically relevant covariates.

Missing data were handled according to established statistical principles. In general, datasets with <5% missing values were considered unlikely to bias inference and were analysed without imputation. For higher levels of missingness, appropriate statistical techniques—such as single or multiple imputation—were employed to ensure accuracy and minimize bias in trial results.

## Results

Twenty-five centers from 15 cities and 5 major islands of Indonesia's archipelago participated in this study. A total 1,599 AF patients (aged 59 ± 13.47 years old, 52% male) were included. Most patients were between 40 and 65 years of age (57.5%). The proportion of paroxysmal, persistent, long-standing persistent and permanent AF were 37%, 22%, 17% and 24% respectively.

Hypertension and chronic renal disease (CKD) are the two most frequent co-morbidities in our AF population. Notably, the definition of CKD in this study is simply based on Creatinine plasma of more than 1.2 mg/dL.

The overall mean CHA_2_DS_2_-VASc score was 2.43, higher in women (2.73) than in men (2.15). Time in therapeutic range (TTR) was only 30.1%. The majority of patients have low bleeding risk as shown by HASBLED score of ≤2 in 95% of patients. The baseline characteristics of all patients are presented in Table 1.

**Table 1:** Baseline characteristics.

Variable	N available	Value [mean ± SD or *n* (%)]
Age (years)	1,599	59 ± 13.5
Age groups (n, %)	1,599	
<40 years		144 (9%)
40–65 years		919 (57.5%)
66–80 years		482 (30.1%)
>80 years		54 (3.4%)
Sex (n, %)	1,599	
Male		835 (52.2%)
Female		764 (47.8%)
AF type (n, %)	1,599	
Paroxysmal		520 (32.5%)
Persistent		375 (23.5%)
Long-standing persistent		286 (17.9%)
Permanent		418 (26.1%)
Hypertension (n, %)	1,582	
Yes		710 (44.4%)
No		872 (54.5%)
Serum creatinine (mean ± SD)	1,258	1.52 ± 4.31
Chronic kidney disease (n, %)	1,258	
Cr ≤ 1.2 mg/dL		872 (54.5%)
Cr > 1.2 mg/dL		386 (24.1%)
CHA_2_DS_2_-VASc score (mean ± SD)	1,599	2.50 ± 1.52
Male		2.20 ± 1.49
Female		2.82 ± 1.5
HAS-BLED score	1,599	
≤2 (n, %)		1,511 (94.5%)
≥3 (n, %)		88 (5.5%)
INR values (mean ± SD)	1,599	2.44 ± 0.6
Time in Therapeutic Range (TTR)	1,599	30.1%;
Left Ventricular Ejection Fraction (n, %)	1,591	
Preserved (LVEF ≥55%)		904 (56.5%)
Reduced (LVEF <55%)		687 (43%)
Left atrial dimension (n, %)	1,593	
Normal (≤40 mm)		852 (53.5%)
Enlarged (>40 mm)		741 (46.3%)
Valvular Heart Disease (n, %)	244	
Moderate Mitral Stenosis (MS)		128 (52.5%)
Severe MS		116 (47.5%)

Denominators reflect the number of patients with available data for each variable, Missing data: LVEF (11%), LAD (14%), INR and TTR were calculable in all patients with ≥3 INR measurements during follow-up.

Rheumatic heart disease (RHD) remains prevalent in Indonesia. In our cohort, 15% of patients (*n* = 244) presented with moderate to severe rheumatic mitral stenosis. Subgroup analysis of patients with valvular heart disease demonstrated that these individuals were younger (48.31 ± 11.34 vs. 60.61 ± 12.95 years, *p* < 0.001), had higher left ventricular ejection fraction (57.00 ± 10.96 vs. 53.78 ± 14.02, *p* = 0.001), and exhibited larger left atrial dimensions (44.72 ± 28.01 vs. 39.50 ± 22.72 mm, *p* = 0.001) compared with non-valvular AF44) patients. No statistically significant differences were observed in INR values or TTR achievement between valvular and non-valvular AF groups. The incidence rate of events per 100 patient-year are shown in Table 2. The overall stroke, major bleeding, and death were 1.32, 1.51 and 3.15 respectively.

**Table 2:** Events incidence rate per 100 patients-year.

Events	Stroke	Major bleeding	Death
Total Event	35	40	81
Total Patient-Year	2,651	2,641	2,569
Incidence rate per 100 patient-year	1.32	1.51	3.15

**Figure 1:**
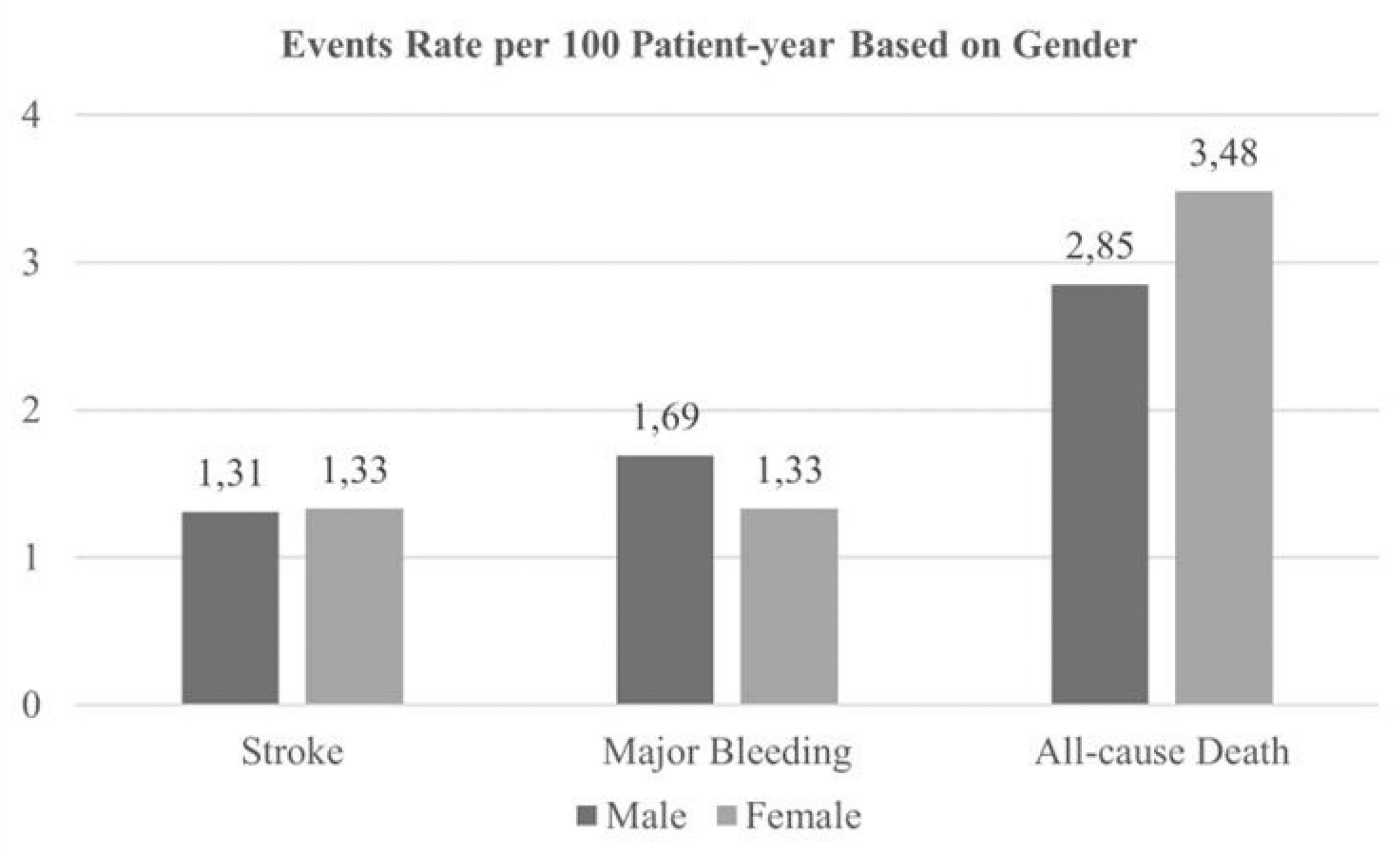
Events rate per 100 patient-year based on gender. No significant difference of events rate between men and women.

The types of AF were associated with the occurrence of stroke (*p* value = 0.004 with *α* < 0.05) that are 21.4%, 14.3%, 28.6% and 35.7% of paroxysmal, persistent, long-standing persistent and permanent AF respectively. Most patients with stroke having a normal LV systolic function (LVEF ≥ 55%) and normal left atrial dimension (LAD) measured from long axis view (LAD ≤ 40 mm). However, there was no statistical correlation between both left ventricle ejection fraction (LVEF) and LAD with stroke event (*p* value of 0.13 and 0.23, respectively). Based on gender, the events rate was not significant between man and woman. Stroke incidence per 100 patient-year were 1.31 and 1.33 in man and woman respectively (*p* value of 0.97). Major bleeding incidence per 100 patient-year were 1.69 and 1.33 in man and woman respectively (*p* value of 0.45). Death incidence per 100 patient-year were 2.85 and 3.48 in man and woman respectively (*p* value of 0.37) (Figure 1).

**Figure 2:**
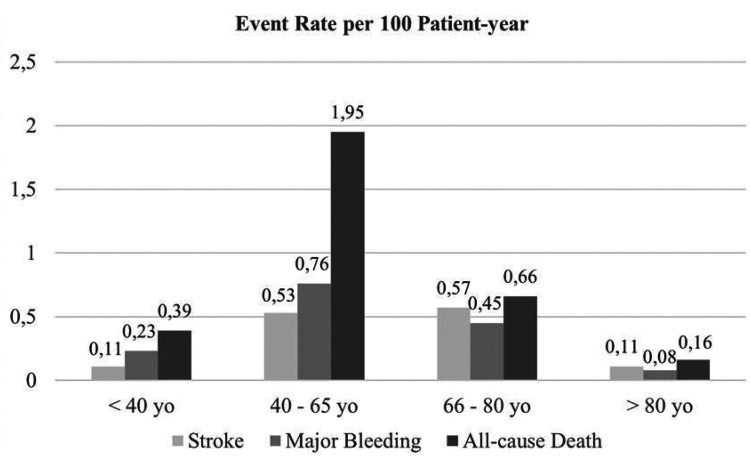
Events rate per 100-patient year. yo, years old.

Figure 2 shows events rate classified by age groups. Stroke incidence per 100 patient-year were 0.11, 0.53, 0.57, 0.11 for <40, 40–65, 66–80, and >80 years old respectively. Major bleeding incidence per 100 patient-year were 0.23, 0.76, 0.45, 0.08 for <40, 40–65, 66–80, and >80 years old respectively. Mortality incidence per 100 patient-year were 0.39, 1.95, 0.66, 0.16 for <40, 40–65, 66–80, and >80 years old respectively.

**Figure 3:**
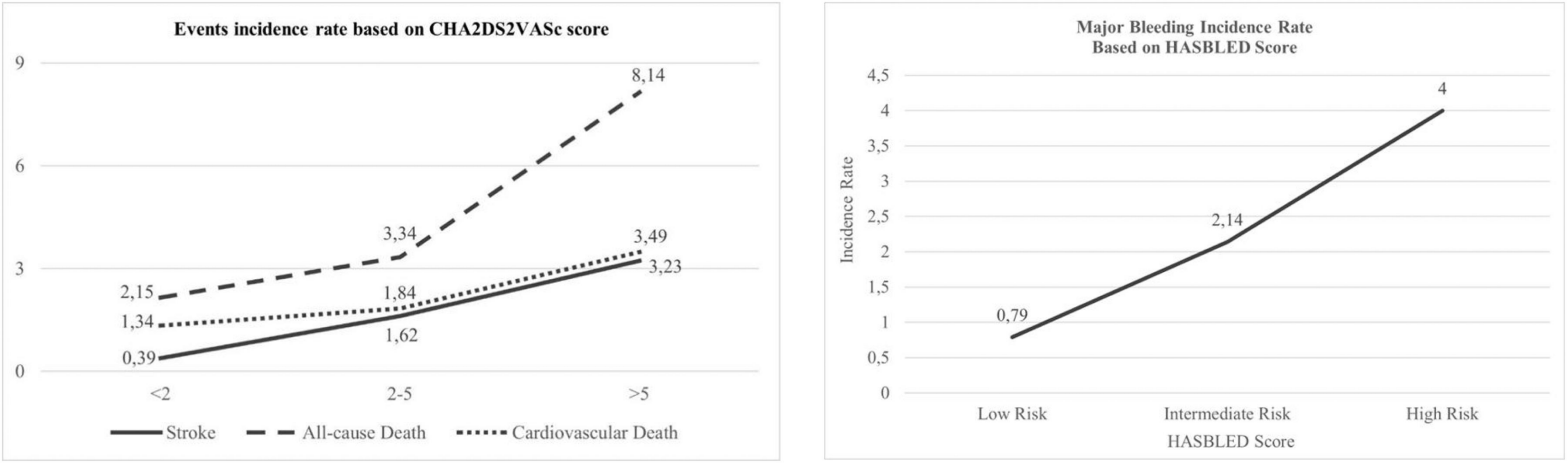
Events rate per 100 patient-years based on CHA_2_DS_2_-VASc score (left) and HASBLED score (right).

We categorized events rate based on CHA_2_DS_2_-VASc score: low risk (score of less than 2), high risk (score of 2–5) and very high risk (score more than 5). The higher the CHA_2_DS_2_-VASc score the higher the event rate. Incidence of ischemic stroke per 100 patient-year were 0.4, 1.6, and 3.2 for low, high and very high-risk patient respectively. Incidence of death per 100 patient-year were 2.2, 3.3, and 8.1 for low, high and very high-risk patient respectively (Figure 3).

Table 3 shows age-stratified event rates with corresponding 95% confidence interval. Individuals aged 40–65 years experienced substantially higher risks, particularly for all-cause death (1.95; 95% CI 1.44–2.57), alongside elevated rates of stroke (0.53; 95% CI 0.29–0.89) and major bleeding (0.76; 95% CI 0.46–1.17). Interestingly, the 66–80-year group showed comparable stroke risk (0.57; 95% CI 0.32–0.93) but a lower bleeding rate (0.45; 95% CI 0.23–0.79) and reduced mortality (0.66; 95% CI 0.39–1.06) compared with the 40–65-year cohort, suggesting possible survivor bias attributed to COVID-19 infection. Patients older than 80 years had the lowest observed event rates across all categories—stroke 0.11 (95% CI 0.02–0.33), bleeding 0.08 (95% CI 0.01–0.27), and mortality 0.16 (95% CI 0.04–0.40)—likely reflecting the small sample size.

**Table 3:** Events rate categorized by ages group.

Ages	Event rate (95% CI)		
	Stroke	Major bleeding	All-cause death
<40 yo	0,11 (0,02–0,33)	0,23 (0,08–0,49)	0,39 (0,19–0,72)
40–65 yo	0,53 (0,29–0,89)	0,76 (0,46–1,17)	1,95 (1,44–2,57)
66–80 yo	0,57 (0,32–0,93)	0,45 (0,23–0,79)	0,66 (0,39–1,06)
>80 yo	0,11 (0,02–0,33)	0,08 (0,01–0,27)	0,16 (0,04–0,40)

The major bleeding event rate per 100 patient-years was categorized based on the HAS-BLED score, in which a score of 0 indicates low risk, 1–2 indicates moderate risk, and ≥3 indicates high risk. The major bleeding event rates were 0.79, 2.14, and 4 for low, moderate and high-risk patients respectively (Figure 3).

**Figure 4:**
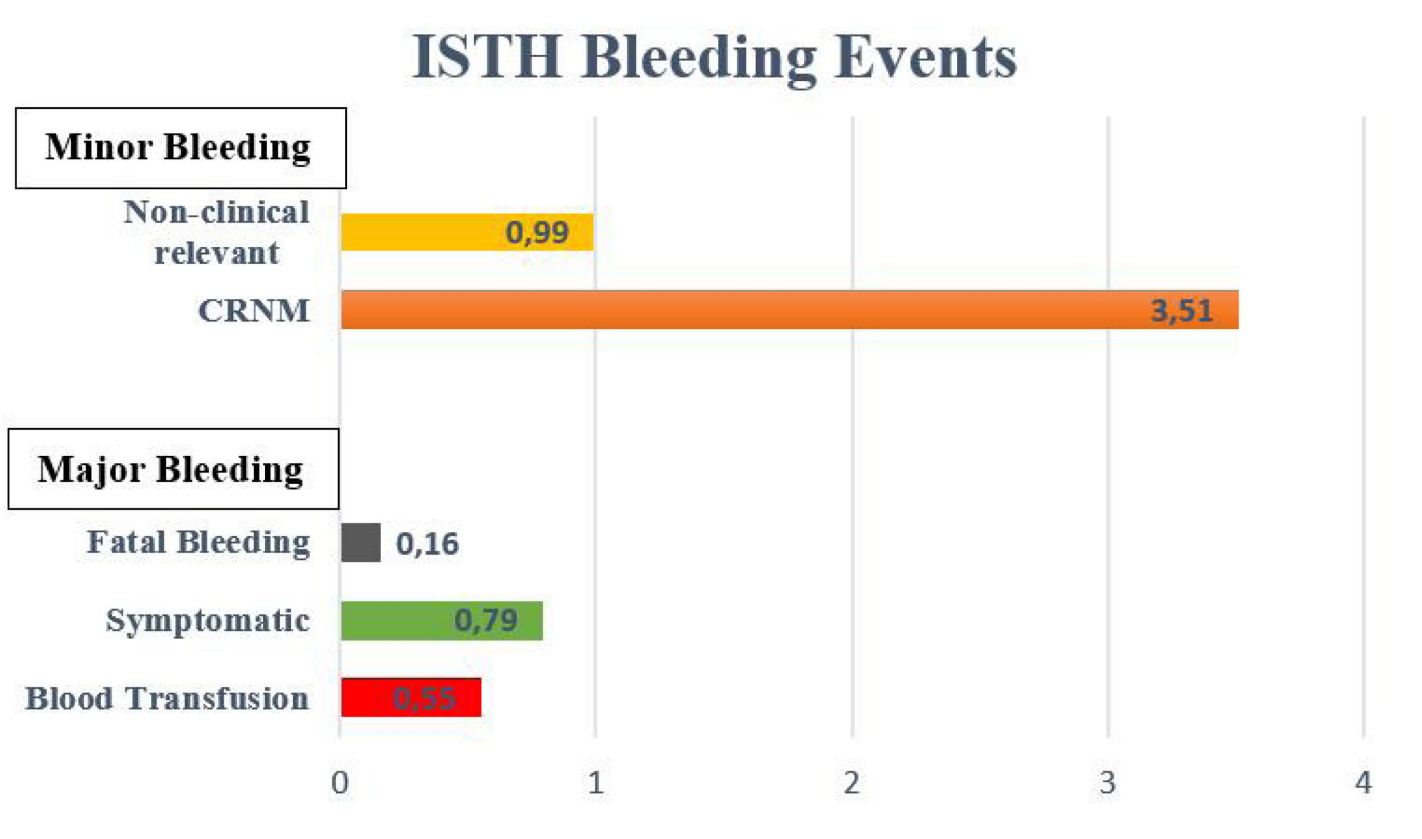
Major and minor bleeding events based on ISTH bleeding classifications. Minor bleeding comprised of non-clinical relevant minor bleeding and clinical-relevant non-major (CRNM) bleeding; major bleeding comprised of fatal bleeding, symptomatic bleeding in critical area organ, and bleeding that need blood transfusion. The graph represents absolute event rates.

The bleeding event rate was 6% per year. Major bleeding occurred in only 1.5% and minor bleeding was 4.5% of all participated patients. Figure 4 shown the breakdown of major and minor bleeding events.

The mortality rate per 100 patients-year were 3.15 and 44.8 permilles in male and female patients respectively. The productive age groups of more than 40–65 years old are patients with the highest mortality rate.

The causes of death were variable with the most common was non cardiovascular death (24%) (Table 4). Notably that the study was conducted at the end period of Covid-19 pandemic which attributed to higher non-cardiovascular deaths. Among the 10 non-cardiovascular deaths observed, six were attributable to confirmed COVID-19 infection, while the remaining four were due to pneumonia suspected to be COVID-19.

**Table 4:** Cause of death.

Cause of death	*N* = 42
Sudden Cardiac Death	7 (17%)
Heart Failure	2 (5%)
Non-Cardiovascular Death	10 (24%)
Valve Surgery	3 (7%)
Stroke Hemorrhagic	4 (10%)
Stroke non-hemorrhagic	2 (5%)
Cardiogenic Shock	6 (14%)
Undetermined	8 (19%)

**Figure 5:**
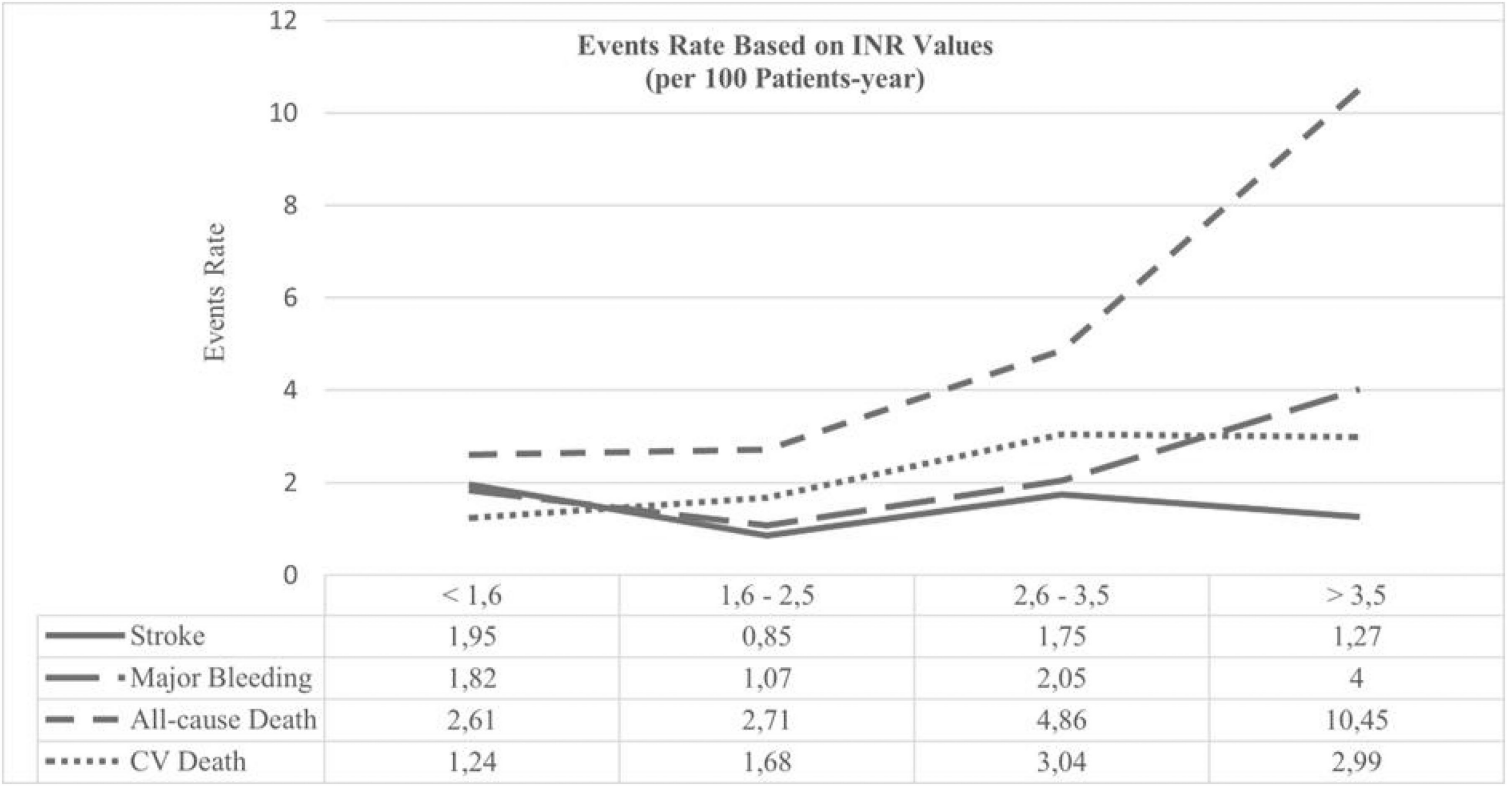
Events rate per 100 patient-years based on INR values.

We conducted analysis of events rate per 100 patient-years based on average INR values. Interestingly, we found that, in our AF population, the average INR of 1.6–2.5 had the lowest stroke, major bleeding, and death risk (Figure 5). To minimize bias from excess non-cardiovascular deaths related to COVID-19, we performed an analysis of cardiovascular death, which consistently showed a lower incidence of the events at the average INR of 1.6–2.5.

We analyzed baseline characteristics stratified by INR group to evaluate potential confounding (Supplementary 1). The cohort represented a relatively young AF population (mean age 59 years, with only 3.4% aged >80). Females had higher CHA_2_DS_2_-VASc scores overall, yet INR stratification revealed that women were disproportionately represented in higher INR categories. Paroxysmal AF clustered within the 1.6–2.5 INR group, while hypertension was strongly associated with INR strata (*p* < 0.001). Reduced LVEF was more common in lower INR categories, whereas preserved LVEF predominated in the therapeutic range. Without adjustment, these imbalances could distort the apparent protective effect of the lower average INR (1.6–2.5). In contrast, renal function, left atrial size, and valvular disease did not differ significantly across strata. To determine whether the observed benefit of lower INR targets was independent of baseline differences, we performed logistic regression analysis of baseline characteristics associated with composite outcomes (stroke, bleeding, and death) (Table 5).

**Table 5:** Logistic regression analysis association with composite outcome.

Variable	Bivariat (Unadjusted) OR (95% CI) *p*-value		Multivariat (Adjusted) aOR (95% CI) *p*-value	
Ages (years)	0.99 (0.98–1.01)	0.65	-	-
Ages Group				
<40 years (Ref)	1.00	0.63	-	-
40–65 years	0.80 (0.45–1.44)			
66–80 years	0.77 (0.41–1.45)			
>80 years	1.28 (0.49–3.33)			
Sex				
Male (Ref)	1.00	0.41	-	-
Female	1.15 (0.81–1.63)			
AF Type				
Paroxysmal (Ref)	1.00	0.04	1.00	0.18
Persistent	1.70 (1.06–2.72)		1.53 (0.94–2.47)	
Long-standing persistent	1.80 (1.09–2.97)		1.61 (0.96–2.70)	
Permanent	1.14 (0.69–1.89)		1.14 (0.68–1.91)	
INR Mean	1.22 (1.03–1.45)	0.01		
Average INR Group				
<1.6	1.42 (0.95–2.13)	0.001	1.41 (0.93–2.12)	0.001
1.6–2.5 (Ref)	1.00		1.00	
2.6–3.5	2.19 (1.34–3.57)		2.24 (1.36–3.69)	
>3.5	3.63 (1.71–7.68)		3.60 (1.67–7.77)	
Hypertension				
No (Ref)	1.00	0.61	-	-
Yes	0.91 (0.64–1.29)			
Diabetes Mellitus				
No (Ref)	1.00	0.27	-	-
Yes	1.26 (0.82–1.94)			
Chronic Kidney Disease				
≤1.2 mg/dL (Ref)	1.00	0.60	-	-
>1.2 mg/dL	1.09 (0.77–1.54)			
CHA_2_DS_2_-VASc Score	1.28 (1.15–1.43)	<0.001	1.24 (1.10–1.39)	<0.001
HAS-BLED score				
≤2 (Ref)	1.00	0.002	1.00	0.10
≥3	2.46 (1.39–4.36)		1.68 (0.89–3.15)	
Left Ventricular Ejection Fraction				
LVEF < 55%	1.39 (0.98–1.97)	0.057	1.24 (0.86–1.77)	0.23
LVEF ≥ 55% (Ref)	1.00		1.00	
LA Dimension				
≤40 mm (Ref)	1.00	0.11	1.00	0.17
>40 mm	1.32 (0.94–1.87)		1.28 (0.89–1.83)	
Valvular Heart Disease				
No Mitral Stenosis (Ref)	1.00	0.79	-	-
Moderate Mitral Stenosis	1.17 (0.64–2.14)			
Severe Mitral Stenosis	0.87 (0.43–1.76)			

After multivariable adjustment, the following findings remained noteworthy: (1) AF Type: Compared with paroxysmal AF, persistent and long-standing persistent AF demonstrated higher odds of adverse composite outcomes [aOR = 1.53 (95% CI 0.94–2.47) and aOR = 1.61 (95% CI 0.96–2.70), respectively], though these associations lost statistical significance after adjustment (*p* = 0.18). Permanent AF showed no meaningful difference [aOR = 1.14 (95% CI 0.68–1.91)]. (2) Average INR Group: average INR strata were strongly associated with outcomes (overall *p* = 0.001). Compared with the reference range (1.6–2.5), both supratherapeutic and subtherapeutic average INR values increased risk: INR < 1.6: aOR = 1.41 (95% CI 0.93–2.12), INR 2.6–3.5: aOR = 2.24 (95% CI 1.36–3.69), INR > 3.5: aOR = 3.60 (95% CI 1.67–7.77). This indicates a U-shaped relationship, where both low and high INR values are associated with increased adverse events. (3) CHA_2_DS_2_-VASc Score: Each one-point increase in CHA_2_DS_2_-VASc score was independently associated with 24% higher odds of composite events [aOR = 1.24 (95% CI 1.10–1.39), *p* < 0.001], confirming its strong predictive value for thromboembolic risk. (4) HAS-BLED Score: Although high bleeding risk (≥3) was significant in unadjusted analysis [OR = 2.46 (95% CI 1.39–4.36), *p* = 0.002], the association attenuated after adjustment [aOR = 1.68 (95% CI 0.89–3.15), *p* = 0.10], suggesting confounding by other clinical factors. (5) Left Ventricular Function and Atrial Size: Reduced LVEF < 55% and enlarged LA > 40 mm showed modest, non-significant trends toward higher risk [aOR = 1.24 (95% CI 0.86–1.77) and aOR = 1.28 (95% CI 0.89–1.83), respectively].

**Figure 6:**
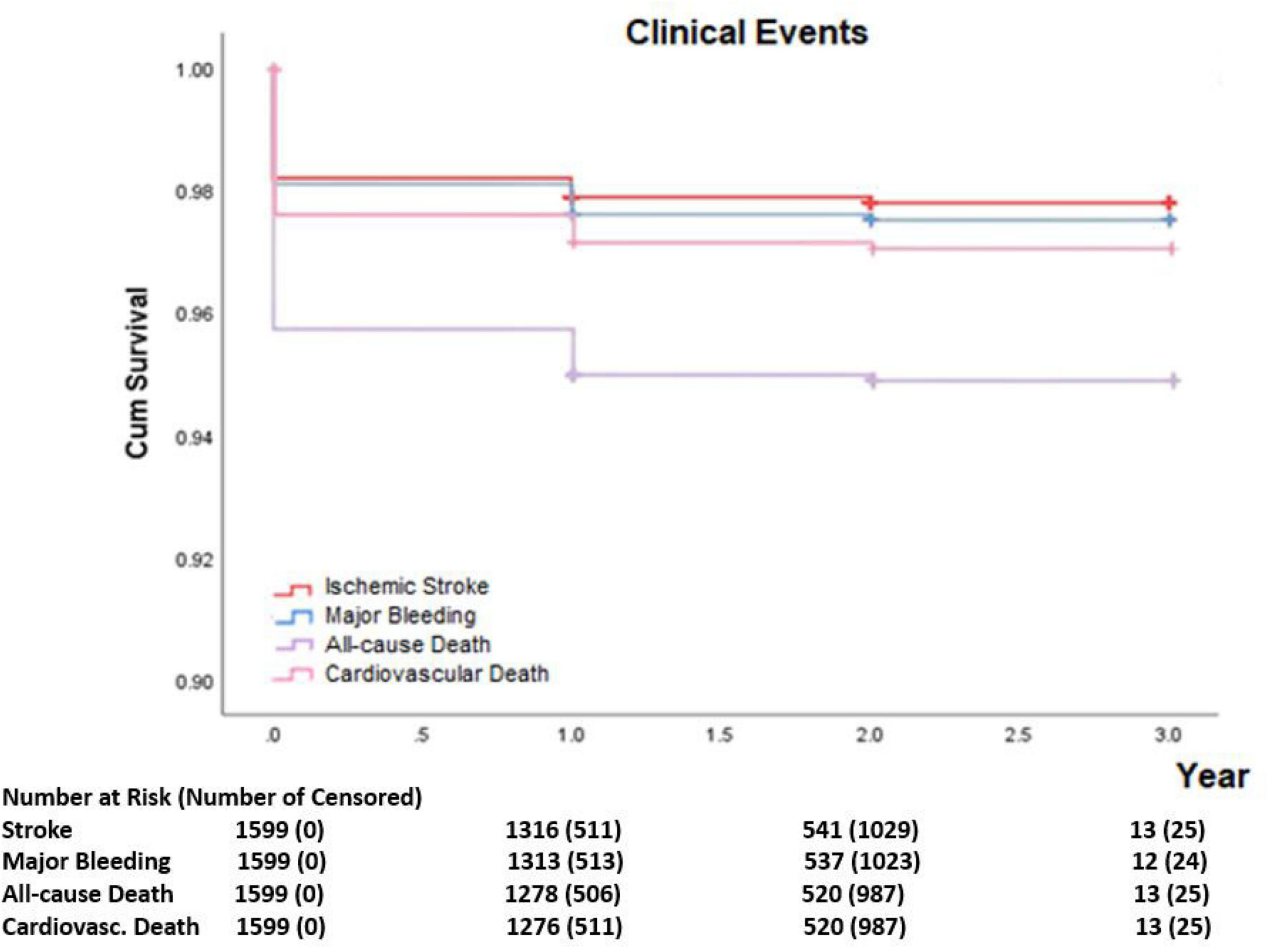
Kaplan–Meier curve of clinical events (ischemic stroke, major bleeding, cardiovascular- and all-cause death).

The Kaplan–Meier survival analysis illustrates the cumulative survival probabilities of AF patients over a three-year period following warfarin treatment. The curves were flat showing that three-year probability of free from stroke, major bleeding and cardiovascular death was more than 95% (Figure 6).

## Discussion

This multicenter cohort provides the first large-scale prospective data on atrial fibrillation patients treated with warfarin in Indonesia. Consistent with prior Asian studies (24, 25) we observed poor anticoagulation quality under the predefined target INR of 2.0–3.0, with a mean TTR of only 30.1%. This finding underscores the challenge of maintaining therapeutic anticoagulation in resource-limited settings where warfarin remains the only reimbursed oral anticoagulant.

Post hoc analyses demonstrated that patients who maintained an average INR of 1.6–2.5 experienced lower rates of stroke, major bleeding, and mortality compared with those in higher or lower INR strata. Importantly, these ranges represent observed average INR categories rather than prospectively applied therapeutic targets. Physicians in our study were instructed to manage patients within the standard INR range of 2.0–3.0, and dose adjustments were made accordingly. Thus, the apparent improvement in outcomes among patients with average INR 1.6–2.5 reflects an association rather than a predefined treatment strategy. Despite these limitations, our findings are hypothesis-generating and suggest that lower average INR values may be associated with improved safety and efficacy in Asian populations. This observation aligns with prior reports of increased bleeding risk among Asians at standard anticoagulation intensities. This aligns with findings from the J-RHYTHM registry, which showed that an INR of 1.6–2.6 is safe and effective for Japanese patients (26). Prior studies have suggested lower INR targets for Asian populations (27–29) though most were retrospective or small-scale (30, 31). Current AF guidelines recommend an INR range of 2.0–3.0, based on Western population data (32, 33). Our study, being a larger prospective cohort, reinforces the need for ethnic-specific INR targets, particularly for Indonesians. However, prospective trials in which physicians are instructed to manage patients within a lower INR range are required to validate whether such a strategy can be recommended. In parallel, expanding access to direct oral anticoagulants (DOACs) remains a critical priority, given their superior efficacy, safety, and ease of use compared with warfarin.

This study revealed that majority of Indonesian AF patients are between 40 and 65 years old, an age group critical to economic productivity. Stroke in this population has profound socioeconomic consequences, as 98.8% of Indonesians over 60 remain part of the workforce (34). In contrast, AF prevalence in Western populations peaks in individuals over 65 (35). Bhonsale et al. reported that AF patients under 65 have significantly worse survival than those without AF, with hazard ratios of 1.5 in men and 2.4 in women under 50, and 1.3 and 1.7, respectively, for ages 50–65 (36). Given these factors, better stroke prevention strategies are critical for this age group.

Despite a low TTR, stroke and major bleeding rates in our cohort were unexpectedly low (1.32 and 1.51 per 100 patient-years, respectively). This apparent paradox may be explained by the relatively younger AF population and the lower mean CHA_2_DS_2_-VASc score observed in Indonesia. The mean score of 2.43 in our cohort is notably lower than those reported in other Asian registries, such as Thailand's COOL-AF (3.1) and Taiwan's National Health Insurance Research Database (median of 3.0) (37, 38). These differences likely reflect a younger age distribution, lower prevalence of hypertension and diabetes, and possibly earlier detection or inclusion of paroxysmal AF cases in Indonesian tertiary centers. In contrast, Western cohorts—including the FinACAF registry (2.6) and the Birmingham European cohorts (2.8) (39, 40) demonstrate similar or slightly higher mean scores, suggesting that Indonesian AF patients align more closely with Western risk profiles than with East Asian populations. This finding supports the hypothesis that stroke risk stratification thresholds may differ regionally, and that Indonesian AF patients may reach anticoagulation thresholds at lower CHA_2_DS_2_-VASc scores compared with Western populations, but higher than expected for truly low-risk groups. Furthermore, our data suggest that Indonesian AF patients may require a lower INR target to achieve effective anticoagulation. Supporting this, real-world data from Korea reported substantially higher stroke (12.9–13.7%) and bleeding (13.8–14.7%) rates among warfarin-treated AF patients (41). Sustained and dedicated efforts are required to improve TTR achievement to levels exceeding 70%. Patients that spent at least 70% of their time in therapeutic range had a 79% reduced risk of stroke compared with patients with ≤30% of TTR (42). Research suggests where patients have a greater knowledge of warfarin therapy, INR values are more often within the target therapeutic range (43). A simple one-off behavioural education session, which significantly improves adherence to warfarin as evidenced by greater TTR compared with usual care. Improving patient understanding surrounding AF, treatment necessity and stroke risk reduction, facilitates informed decisions about the management of their condition and treatment and can also make a significant difference to long-term adherence (44). Finally, the meta-analysis by Carmo et al. demonstrated that the superiority of DOACs over warfarin diminishes once TTR exceeds 70%, reinforcing the view that well-managed warfarin therapy remains a viable option (45).

Stroke incidence correlated with AF type in our cohort, with persistent and permanent AF associated with higher risk. The ROCKET-AF trial found similar results, with persistent AF patients having increased stroke/systemic embolism (2.18 vs. 1.73 events per 100 patient-years, *p* *=* *0.048*) and all-cause mortality (4.78 vs. 3.52, *p* *=* *0.006*) (46). Notably, most stroke patients in our study had preserved left ventricular function and normal left atrial size, indicating that early AF detection and intervention could be beneficial. The EAST-AFNET 4 trial demonstrated that early rhythm control significantly improves cardiovascular outcomes (47), reinforcing the importance of proactive rhythm management, including ablation therapy.

Our Kaplan–Meier analysis showed that three-year survival free from stroke, major bleeding, and cardiovascular death exceeded 95%. However, mortality rates were influenced by non-cardiovascular deaths, particularly during the late COVID-19 pandemic period. The high prevalence of AF in Indonesia, especially among younger adults, aligns with findings from Wong et al., who reported an increasing AF burden across Asia (48). While DOACs provide superior efficacy and safety (49, 50), they remain inaccessible under Indonesia's National Health Insurance due to cost constraints. Given the economic impact of AF-related stroke, policymakers should consider selective DOAC implementation, particularly for high-risk, working-age patients. Until then, optimizing warfarin therapy with an standard INR target and good TTR remain a practical and cost-effective strategy for stroke prevention in Indonesia.

## Limitations

This study has several important limitations. First, all patients were prospectively managed with a predefined *target INR* of 2.0–3.0, and physician behavior was guided accordingly. The *post hoc* categorization of patients into average INR ranges (e.g., 1.6–2.5, 2.6–3.5) reflects observed exposure rather than a true modification of the therapeutic target. Second, our analysis did not isolate a group with average INR 2.0–3.0, which limits direct comparison with the current standard practice. As such, prospective validation is warranted to recommend a lower target INR. Third, the use of average INR over the follow-up period may obscure the temporal relationship between INR values and clinical events, which are more likely to be influenced by INR levels at or near the time of the event. Finally, the study was conducted during the late COVID-19 pandemic period, which may have influenced overall mortality rates, particularly for non-cardiovascular deaths.

## Conclusion

In this large, multicenter cohort of Indonesian atrial fibrillation patients, anticoagulation with warfarin under the predefined target INR of 2.0–3.0 was associated with poor time in therapeutic range (30.1%) and suboptimal outcomes. *Post hoc* analyses demonstrated that patients who maintained an average INR of 1.6–2.5 experienced lower rates of stroke, major bleeding, and mortality compared with those in higher or lower INR strata. However, these findings represent observational associations rather than prospectively applied therapeutic targets.

The relatively younger age of our cohort, combined with a lower mean CHA_2_DS_2_-VASc score compared with Western populations, may partly explain the lower-than-expected event rates. Nonetheless, the socioeconomic burden of AF-related stroke in working-age Indonesians underscores the need for optimized anticoagulation strategies. While direct oral anticoagulants (DOACs) offer superior efficacy and safety, their limited accessibility under Indonesia's National Health Insurance necessitates pragmatic approaches to warfarin management. Tailoring INR targets to local populations and healthcare resources may represent a cost-effective interim strategy, while selective DOAC implementation in high-risk patients could further improve outcomes.

Future studies should validate these findings in larger, diverse cohorts, with prespecified lower INR targets and standardized monitoring protocols.

## Data Availability

The raw data supporting the conclusions of this article will be made available by the authors, without undue reservation.

## References

[1] Nguyen TN Hilmer SN Cumming RG. Review of epidemiology and management of atrial fibrillation in developing countries. Int J Cardiol. (2013) 167:2412–20. 10.1016/j.ijcard.2013.01.184 23453870

[2] Chiang CE Zhang S Tse HF Teo WS Omar R Sriratanasathavorn C. Atrial fibrillation management in Asia: from the Asian expert forum on atrial fibrillation. Int J Cardiol. (2013) 164:21–32. 10.1016/j.ijcard.2011.12.033 22240753

[3] Chien KL Su TC Hsu HC Chang WT Chen PC Chen MF. Atrial fibrillation prevalence, incidence and risk of stroke and all-cause death among Chinese. Int J Cardiol. (2010) 139:173–80. 10.1016/j.ijcard.2008.10.045 19046608

[4] Tse HF Wang YJ Ahmed Ai-Abdullah M Pizarro-Borromeo AB Chiang CE Krittayaphong R. Stroke prevention in atrial fibrillation–an Asian stroke perspective. Heart Rhythm. (2013) 10:1082–8. 10.1016/j.hrthm.2013.03.017 23501173

[5] Chiang CE Wang KL Lip GY. Stroke prevention in atrial fibrillation: an Asian perspective. Thromb Haemost. (2014) 111:789–97. 10.1160/TH13-11-0948 24500243

[6] Long MJ Jiang CQ Lam TH Xu L Zhang WS Lin JM. Atrial fibrillation and obesity among older Chinese: the Guangzhou biobank cohort study. Int J Cardiol. (2011) 148:48–52. 10.1016/j.ijcard.2009.10.022 19944468

[7] Iguchi Y Kimura K Shibazaki K Aoki J Kobayashi K Sakai K. Annual incidence of atrial fibrillation and related factors in adults. Am J Cardiol. (2010) 106:1129–33. 10.1016/j.amjcard.2010.06.030 20920652

[8] Jeong JH. Prevalence of and risk factors for atrial fibrillation in Korean adults older than 40 years. J Korean Med Sci. (2005) 20:26–30. 10.3346/jkms.2005.20.1.26 15716597 PMC2808570

[9] Yap KB Ng TP Ong HY. Low prevalence of atrial fibrillation in community-dwelling Chinese aged 55 years or older in Singapore: a population-based study. J Electrocardiol. (2008) 41:94–8. 10.1016/j.jelectrocard.2007.03.012 17531253

[10] Lim CW Kasim S Ismail JR Chua NYC Khir RN Zainal Abidin HA. Prevalence of atrial fibrillation in the Malaysian communities. Heart Asia. (2016) 8:62–6. 10.1136/heartasia-2016-010775 27933105 PMC5133392

[11] Yuniadi Y Supit AI Hanafy DA Raharjo SB Hermanto DY Basalamah F. Prevalence of atrial fibrillation based on tertiary hospital survey in Indonesia: a smartphone-based diagnosis. J Arrhythm. (2024) 40:1102–7. 10.1002/joa3.13137 39416241 PMC11474569

[12] Joglar JA Chung MK Armbruster AL Benjamin EJ Chyou JY Cronin EM. 2023 ACC/AHA/ACCP/HRS guideline for the diagnosis and management of atrial fibrillation: a report of the American College of Cardiology/American Heart Association Joint Committee on Clinical Practice Guidelines. Circulation. (2024) 149:e1–156. 10.1161/CIR.0000000000001193 38033089 PMC11095842

[13] Van Gelder IC Rienstra M Bunting KV Casado-Arroyo R Caso V Crijns HJGM. 2024 ESC guidelines for the management of atrial fibrillation developed in collaboration with the European Association for cardio-Thoracic Surgery (EACTS). Eur Heart J. (2024) 45:3314–414. 10.1093/eurheartj/ehae176 39210723

[14] Thompson LE Maddox TM Lei L Grunwald GK Bradley SM Peterson PN. Sex differences in the use of oral anticoagulants for atrial fibrillation: a report from the National Cardiovascular Data Registry (NCDR®). PINNACLE Registry. J Am Heart Assoc. (2017) 6:e005801. 10.1161/JAHA.117.005801 28724655 PMC5586299

[15] Zhu S Li M Wang L Hou L Liu J. Real-world national trends and influencing factors preference of non-vitamin K antagonist oral anticoagulants in China. Front Med (Lausanne). (2023) 10:1258536. 10.3389/fmed.2023.1258536 38076271 PMC10702983

[16] Krittayaphong R Winijkul A Methavigul K Wongtheptien W Wongvipaporn C Wisaratapong T. Risk profiles and pattern of antithrombotic use in patients with non-valvular atrial fibrillation in Thailand: a multicenter study. BMC Cardiovasc Disord. (2018) 18:174. 10.1186/s12872-018-0911-4 30144802 PMC6109333

[17] van Asch CJ Luitse MJ Rinkel GJ van der Tweel I Algra A Klijn CJ. Incidence, case fatality, and functional outcome of intracerebral haemorrhage over time, according to age, sex, and ethnic origin: a systematic review and meta-analysis. Lancet Neurol. (2010) 9:167–76. 10.1016/S1474-4422(09)70340-0 20056489

[18] Takahashi H Wilkinson GR Caraco Y Muszkat M Kim RB Kashima T. Population differences in S-warfarin metabolism between CYP2C9 genotype-matched Caucasian and Japanese patients. Clin Pharmacol Ther. (2003) 73:253–63. 10.1067/mcp.2003.26a 12621390

[19] Rieder MJ Reiner AP Gage BF Nickerson DA Eby CS McLeod HL. Effect of VKORC1 haplotypes on transcriptional regulation and warfarin dose. N Engl J Med. (2005) 352:2285–93. 10.1056/NEJMoa044503 15930419

[20] Suriapranata IM Tjong WY Wang T Utama A Raharjo SB Yuniadi Y. Genetic factors associated with patient-specific warfarin dose in ethnic Indonesians. BMC Med Genet. (2011) 12:80. 10.1186/1471-2350-12-80 21639946 PMC3133537

[21] Schulman S Kearon C, Subcommittee on Control of Anticoagulation of the Scientific and Standardization Committee of the International Society on Thrombosis and Haemostasis. Definition of major bleeding in clinical investigations of antihemostatic medicinal products in non-surgical patients. J Thromb Haemost. (2005) 3:692–4. 10.1111/j.1538-7836.2005.01204.x 15842354

[22] WHO Expert Committee on Biological Standardization. Requirements for thromboplastins and plasma used to control oral anticoagulant therapy (requirements for biological substances no.30, revised 1982). In: WHO Expert Committee on Biological Standardization. Thirty-Third Report. Annex 3, WHO Technical Report Series, no. 687. Geneva: World Health Organization (1983). p. 97–123.

[23] Rosendaal FR Cannegieter SC van der Meer FJ Briët E. A method to determine the optimal intensity of oral anticoagulant therapy. Thromb Haemost. (1993) 69(3):236–9. 10.1055/s-0038-1651587 8470047

[24] Cheng CY Lian TY Zhu XJ Virdone S Sun K Camm J. Atrial fibrillation outcomes in patients from Asia and non-Asia countries: insights from GARFIELD-AF. Open Heart. (2025) 12(1):e003109. 10.1136/openhrt-2024-003109 39914996 PMC11804197

[25] Tse HF Teo WS Siu CW Chao TF Park HW Shimizu W. Prognosis and treatment of atrial fibrillation in Asian cities: 1-year review of the Asia-Pacific Heart Rhythm Society Atrial Fibrillation Registry. EP Europace. (2022) 24(12):1889–98. 10.1093/europace/euab327 35025986

[26] Inoue H Okumura K Atarashi H Yamashita T Origasa H Kumagai N. Target international normalized ratio values for preventing thromboembolic and hemorrhagic events in Japanese patients with non-valvular atrial fibrillation: results of the J-RHYTHM Registry. Circ J. (2013) 77:2264–70. 10.1253/circj.CJ-13-0290 23708863

[27] You JH Chan FW Wong RS Cheng G. Is INR between 2.0 and 3.0 the optimal level for Chinese patients on warfarin therapy for moderate-intensity anticoagulation? Br J Clin Pharmacol. (2005) 59:582–7. 10.1111/j.1365-2125.2005.02361.x 15842557 PMC1884850

[28] Suzuki S Yamashita T Kato T Fujino T Sagara K Sawada H. Incidence of major bleeding complication of warfarin therapy in Japanese patients with atrial fibrillation. Circ J. (2007) 71:761–5. 10.1253/circj.71.761 17457005

[29] Yamaguchi T. Optimal intensity of warfarin therapy for secondary prevention of stroke in patients with nonvalvular atrial fibrillation: a multicenter, prospective, randomized trial. Japanese Nonvalvular Atrial Fibrillation-Embolism Secondary Prevention Cooperative Study Group. Stroke. (2000) 31:817–21. 10.1161/01.STR.31.4.817 10753981

[30] JCS Joint Working Group. Guidelines for pharmacotherapy of atrial fibrillation (JCS 2008): digest version. Circ J. (2010) 74:2479–500. 10.1253/circj.CJ-88-0001 20962419

[31] Yasaka M Minematsu K Yamaguchi T. Optimal intensity of international normalized ratio in warfarin therapy for secondary prevention of stroke in patients with non-valvular atrial fibrillation. Intern Med. (2001) 40:1183–8. 10.2169/internalmedicine.40.1183 11813841

[32] Hindricks G Potpara T Dagres N Arbelo E Bax JJ Blomström-Lundqvist C. 2020 ESC guidelines for the diagnosis and management of atrial fibrillation developed in collaboration with the European Association for Cardio-Thoracic Surgery (EACTS): the task force for the diagnosis and management of atrial fibrillation of the European Society of Cardiology (ESC) developed with the special contribution of the European Heart Rhythm Association (EHRA) of the ESC. Eur Heart J. (2021) 42:373–498. 10.1093/eurheartj/ehaa612 32860505

[33] Joglar JA Chung MK Armbruster AL Benjamin EJ Chyou JY Cronin EM. 2023 ACC/AHA/ACCP/HRS guideline for the diagnosis and management of atrial fibrillation: a report of the American College of Cardiology/American Heart Association Joint Committee on Clinical Practice Guidelines. J Am Coll Cardiol. (2024) 83:109–279. 10.1016/j.jacc.2023.08.017 38043043 PMC11104284

[34] Biro Pusat Statistik (BPS). Survei angkatan kerja nasional (sakernas). BPS. (2024).

[35] Lippi G Sanchis-Gomar F Cervellin G. Global epidemiology of atrial fibrillation: an increasing epidemic and public health challenge. Int J Stroke. (2021) 16:217–21. 10.1177/1747493019897870 31955707

[36] Bhonsale A Zhu J Thoma F Koscumb S Kancharla K Voigt A. Mortality, hospitalization, and cardiac interventions in patients with atrial fibrillation aged <65 years. Circ Arrhythm Electrophysiol. (2024) 17:e012143. 10.1161/CIRCEP.123.012143 38646831 PMC11111318

[37] Krittayaphong R Apiyasawat S Methavigul K Komoltri C Lip GYH. Prediction of ischemic stroke by the CHA_2_DS_2_-VA score in an Asian population: a report from the prospective nationwide COOL-AF registry. Heart Rhythm. (2025) 22(9):e658–67. 10.1016/j.hrthm.2025.04.062 40324512

[38] Chao TF Lip GYH Liu CJ Tuan TC Chen SJ Wang KL. Validation of a modified CHA_2_DS_2_-VASc score for stroke risk stratification in Asian patients with atrial fibrillation. A nationwide cohort study. Stroke. (2016) 47:2462–9. 10.1161/STROKEAHA.116.013880 27625386

[39] Teppo K Lip GYH Airaksinen KEJ Halminen O Haukka J Putaala J. Comparing CHA_2_DS_2_-VA and CHA_2_DS_2_-VASc scores for stroke risk stratification in patients with atrial fibrillation: a temporal trends analysis from the retrospective Finnish AntiCoagulation in Atrial Fibrillation (FinACAF) cohort. Lancet Reg Health Eur. (2024) 43:100967. 10.1016/j.lanepe.2024.100967 39171253 PMC11337097

[40] Mei DA Romiti GF Vitolo M Imberti JF Mantovani M Corica B. Comparison of CHA_2_DS_2_-VA and CHA_2_DS_2_-VASc scores for predicting residual risk of stroke and adverse outcomes in anticoagulated patients with atrial fibrillation: a report from a European registry. EP Europace. (2025) 27(Suppl 1):euaf085.305. 10.1093/europace/euaf085.305

[41] Bang OY On YK Lee MY Jang SW Han SW Han S. The risk of stroke/systemic embolism and major bleeding in Asian patients with non-valvular atrial fibrillation treated with non-vitamin K oral anticoagulants compared with warfarin: results from a real-world data analysis. PLoS One. (2020) 15:e0242922. 10.1371/journal.pone.0242922. eCollection 2020. 33253294 PMC7703907

[42] Gallagher AM Setakis E Plumb JM Clemens A van Staa TP. Risks of stroke and mortality associated with suboptimal anticoagulation in atrial fibrillation patients. Thromb Haemost. (2011) 106:968–77. 10.1160/TH11-05-0353 21901239

[43] Tang EO Lai CS Lee KK Wong RS Cheng G Chan TYK. Relationships between patients’ warfarin knowledge and anticoagulation control. Ann Pharmacother. (2003) 37:34–9. 10.1345/aph.1A198 12503930

[44] Clarkesmith DE Pattison HM Lip GYH Lane DA. Educational intervention improves anticoagulation control in atrial fibrillation patients: the TREAT randomised trial. PLoS One. (2013) 8(9):e74037. 10.1371/journal.pone.0074037 24040156 PMC3767671

[45] Carmo J Ferreira J Costa F Carmo P Cavaco D Carvalho S. Non-vitamin K antagonist oral anticoagulants compared with warfarin at different levels of INR control in atrial fibrillation: a meta-analysis of randomized trials. Int J Cardiol. (2017) 244:196–201. 10.16/j.ijcard.2017.06.004 28679480 10.1016/j.ijcard.2017.06.004

[46] Steinberg BA Hellkamp AS Lokhnygina Y Manesh RP Breithardt G Hankey GJ. Higher risk of death and stroke in patients with persistent vs. paroxysmal atrial fibrillation: results from the ROCKET-AF trial. Eur Heart J. (2015) 36:288–96. 10.1093/eurheartj/ehu359 25209598 PMC4313363

[47] Kirchhof P Camm AJ Goette A Brandes A Eckardt L Elvan A. Early rhythm-control therapy in patients with atrial fibrillation. N Engl J Med. (2020) 383:1305–16. 10.1056/NEJMoa2019422 32865375

[48] Wong CX Tse HF Choi EK Chao TF Inoue K Poppe K. The burden of atrial fibrillation in the Asia-Pacific region. Nat Rev Cardiol. (2024) 21:841–3. 10.1038/s41569-024-01091-1 39322762

[49] Cha MJ Choi EK Han KD Lee SR Lim WH Oh S. Effectiveness and safety of non-vitamin K antagonist oral anticoagulants in Asian patients with atrial fibrillation. Stroke. (2017) 48:3040–8. 10.1161/strokeaha.117.018773 28974629

[50] Wang KL Lip GY Lin SJ Chiang CE. Non-vitamin K antagonist oral anticoagulants for stroke prevention in Asian patients with nonvalvular atrial fibrillation: meta-analysis. Stroke. (2015) 46:2555–61. 10.1161/STROKEAHA.115.009947 26304863 PMC4542566

