# Supplementary material for: The characteristics and clinical outcomes of atrial fibrillation patient in middle income country of Indonesia
Source: Front Cardiovasc Med. 2026 Jul 1;13:1824720. doi: 10.3389/fcvm.2026.1824720 (PMC13369050; doi:10.3389/fcvm.2026.1824720)
Supplement: Supplementary file 1 [file Table1.docx]

**SUPPLEMENT 1**

**Baseline characteristics stratified by Average INR groups**

| Event | Average INR | | | | p-Value |
| --- | --- | --- | --- | --- | --- |
|  | **< 1.6**  **(n=568)** | **1.6 – 2.5**  **(n=773)** | **2.6 – 3.5**  **(n=209)** | **> 3.5**  **(n=49)** |  |
| Ages Group (n, %) |  |  |  |  |  |
| < 40 years | 57 (10%) | 59 (7.6%) | 24 (11.5%) | 4 (8.2%) | 0.330 |
| 40 – 65 years | 318 (56%) | 445 (57.6%) | 131 (62.7%) | 25 (51%) |  |
| 66 – 80 years | 174 (30.6%) | 241 (31.2%) | 49 (23.4%) | 18 (36.7%) |  |
| >80 years | 19 (3.4%) | 28 (3.6%) | 5 (2.4%) | 2 (4.1%) |  |
|  |  |  |  |  |  |
| Sex (n, %) |  |  |  |  | 0.043 |
| Male | 309 (54.4%) | 412 (53.3%) | 92 (44%) | 22 (44.9%) |  |
| Female | 259 (45.6%) | 361 (46.7%) | 117 (56%) | 27 (55.1%) |  |
|  |  |  |  |  |  |
| AF Type (n, %) |  |  |  |  | 0.03 |
| Paroxysmal | 187 (32.9%) | 259 (33.5%) | 61 (29.2%) | 13 (26.5%) |  |
| Persistent | 150 (26.4%) | 165 (21.4%) | 48 (23%) | 12 (24.5%) |  |
| Long-standing persistent | 110 (19.4%) | 117 (15.1%) | 44 (21%) | 15 (30.6%) |  |
| Permanent | 121 (21.3%) | 232 (30%) | 56 (26.8%) | 9 (18.4%) |  |
|  |  |  |  |  |  |
| Hypertension (n, %) |  |  |  |  | < 0.001 |
| Yes | 287 (50.5%) | 337 (43.6%) | 69 (33%) | 17 (34.7%) |  |
| No | 281 (49.5%) | 422 (56.4%) | 137 (67%) | 32 (65.3%) |  |
|  |  |  |  |  |  |
| Chronic Kidney Disease (n, %) |  |  |  |  | 0.092 |
| ≤ 1.2 mg/dL | 329 (58%) | 410 (53%) | 102 (48.9%) | 31 (63.3%) |  |
| > 1.2 mg/dL | 158 (42%) | 162 (47%) | 58 (51.1%) | 8 (36.7%) |  |
|  |  |  |  |  |  |
| HAS-BLED score |  |  |  |  | 0.738 |
| ≤ 2 | 535 (94.2%) | 730 (94.4%) | 198 (94.7%) | 48 (98%) |  |
| ≥ 3 | 33 (5.8%) | 43 (5.6%) | 11 (5.3%) | 1 (2%) |  |
|  |  |  |  |  |  |
| Left Ventricular Ejection Fraction |  |  |  |  | 0.05 |
| LVEF < 55% | 272 (47.9%) | 325 (42%) | 79 (37.8%) | 19 (38.8%) |  |
| LVEF ≥ 55% | 296 (52.1%) | 448 (58%) | 130 (62.2%) | 30 (61.2%) |  |
|  |  |  |  |  |  |
| LA Dimension |  |  |  |  | 0.526 |
| ≤ 40 mm | 298 (52.5%) | 424 (54.9%) | 107 (51.2%) | 23 (59%) |  |
| > 40 mm | 270 (47.5%) | 349 (45.1%) | 102 (48.8%) | 26 (41%) |  |
|  |  |  |  |  |  |
| Valvular Heart Disease (n, %) |  |  |  |  | 0.300 |
| Moderate Mitral Stenosis | 42 (56%) | 52 (46.8%) | 30 (56.6%) | 4 (80%) |  |
| Severe MS | 33 (44%) | 59 (53.2%) | 23 (43.4%) | 1 (20%) |  |
